# Robot-assisted laparoscopic pyeloplasty is a valid option for ureteropelvic junction obstruction repair in adults with congenital renal abnormalities: a case series study

**DOI:** 10.1186/s12894-023-01308-4

**Published:** 2023-08-19

**Authors:** Sarah Razavi, Joshua Babbin, Douglas Dahl

**Affiliations:** 1 Smith institute for urology, Northwell Health, NY Lake Success, USA; 2 Creighton University School of Medicine Omaha, NE Omaha, USA; 3 Massachusetts General Hospital, MA Boston, USA

**Keywords:** Robotic, Pyeloplasty, Renal, Congenital, Abnormality

## Abstract

**Background:**

Congenital renal anomalies are rare but may be associated with obstruction of the ureteropelvic junction. Given the rarity of simultaneous ureteropelvic junction obstruction [UPJO] and renal anomalies in the adult population, there is limited literature on approaching these patients. We report our experience with robotic assisted laparoscopic pyeloplasty for UPJO repair in this subset of patients.

**Methods:**

Data on adult patients with simultaneous congenital renal abnormalities and UPJO who underwent robotic assisted laparoscopic pyeloplasty between 2008 and 2020 was reviewed. Pre-operative data, intraoperative parameters as well as post-operative data including symptom resolution and radiologic findings were recorded.

**Results:**

Ten patients, 4 female and 6 males, with a mean age of 47 years were identified as having simultaneous congenital renal abnormalities and UPJO. Anomalies identified were horseshoe kidney in four patients, duplex kidney with obstruction of one moiety in two patients, malrotated kidney in two patients, and pelvic kidney in two patients. Eight out of ten were symptomatic at presentation with flank pain being the most common symptom. Eight patients underwent robotic pyeloplasty via the dismembered technique, while two underwent robotic Y-V pyeloplasty. With a mean follow up time of 13 months, 8/9 (88%) symptomatic patients enjoyed symptom resolution. Post-op renogram was available for nine patients and showed resolution of obstruction in all patients (100%). One patient developed a urine leak which was managed successfully with drainage.

**Conclusions:**

Robotic assisted laparoscopic pyeloplasty is a safe, feasible and effective surgical approach in management of adult patients with concomitant UPJO and renal anomalies.

## Introduction

Congenital abnormalities of the kidney and urinary tract occur in approximately 3.3–11.1% of the population accounting for about half of all congenital abnormalities [1]. Untreated, they may lead to renal failure in different stages of life [2]. Renal anomalies present unique challenges to adult urologic surgeons. These are due to anatomic variants such as distorted pelvicalyceal architecture, unfamiliar relationships to adjacent structures, and aberrant vasculature [3]. Ureteropelvic junction obstruction (UPJO) is the obstruction of urine flow from the kidney to the proximal ureter. Open surgical correction has a good record for UPJO repair, but minimally invasive surgical techniques have gained popularity [4]. Given the rarity of simultaneous UPJO and renal anomalies in the adult population, there is a paucity of literature on surgical success in this subset of patients. Very few reports addressed minimally invasive treatment of UPJO in adults with upper tract anomalies using laparoscopy [5, 6]. Laparoscopic renal reconstruction requires advanced training and is technically challenging. Robotic assisted laparoscopic pyeloplasty (RAP) offers unique advantages in reconstructive surgery with the advantages of three-dimensional optics and precision in tissue handling and suturing. RAP has gained popularity worldwide for pyeloplasty in pediatric and adult populations [7]. Yet, the studies are less robust using robotic approach in UPJO repair in adults with congenital renal anomalies.

Most efficacy studies of RAP in patients with congenital anomalies and simultaneous UPJO is derived from pediatrics [8, 9]). In this series, we report our experience with robot-assisted laparoscopic pyeloplasty for UPJO repair in adult patients with congenital renal anomalies.

## Materials and methods

After obtaining institutional review board approval, data on adult patients with simultaneous congenital renal abnormalities and UPJO who underwent robotic assisted laparoscopic pyeloplasty between 2010 and 2020 at Massachusetts General Hospital was reviewed. Pre-operative data including demographics, symptoms, renal function, imaging findings, intraoperative parameters as well as post-operative data including symptom resolution and radiologic findings were recorded. Patient were treated for clinical symptoms and/or patients with evidence of obstruction during Tc-99 m mercaptoacetyltriglycine renal scan (MAG3). Success was defined as symptom resolution and/or improvement in radiologic evidence of obstruction. All surgeries were performed by a single surgeon (DD) using the Da Vinci [Intuitive Surgical] surgical system. The study was conducted in accordance with the Declaration of Helsinki.

Statistical analysis was performed with IBM SPSS V27. Continuous variables are reported as mean and standard deviation whereas categorical variables are reported as percentages.

### Surgical technique

**Fig. 1:**
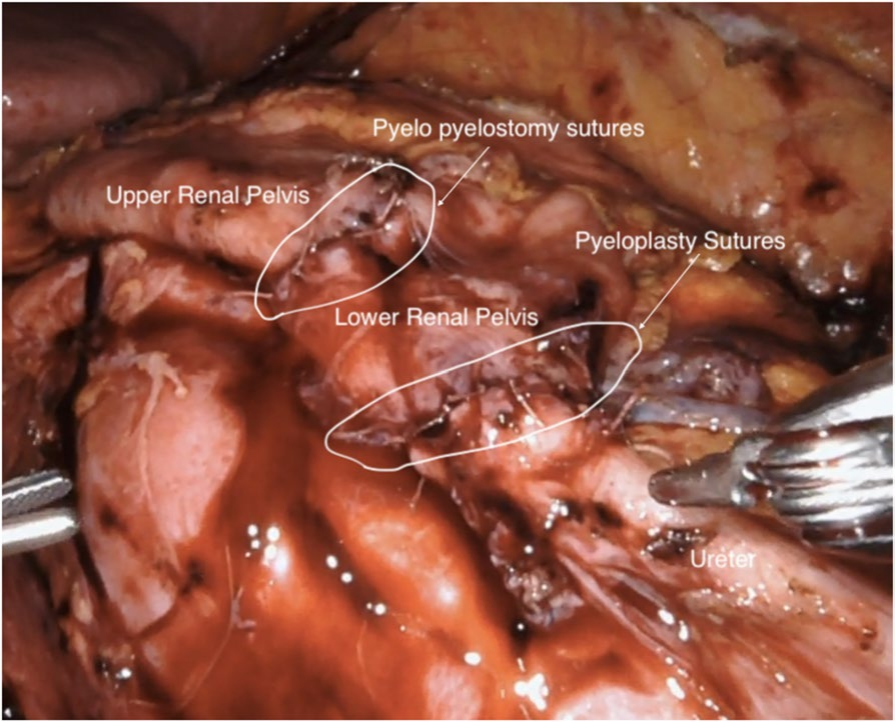
Pyelo pyelotomy/pyeloplasty and relevant sutures

Flexible cystoscopy was used to place a flexible tipped guidewire and open-ended ureteral catheter. Trans-peritoneal abdominal access was gained with the patient in full flank position. Port placement was determined by the estimated level of obstruction. The colon and mesentery were fully mobilized away from the renal pelvis and proximal ureter. Anomalous renal vessels were very carefully dissected away from the ureter and renal pelvis. Thorough mobilization of the renal pelvis was performed to assess the need for reduction of redundant pelvic tissue. Plan of repair was guided by the degree of obstruction, health of tissue, and the ability to achieve tension-free anastomosis of renal pelvis to ureter. The proximal ureter was spatulated. An interrupted anastomosis was performed with 5 − 0 and 4 − 0 polyglycolic suture [Vicryl, Ethicon]. In cases of duplex systems, both renal upper and lower pelvis were adequately dissected from surrounding inflammatory tissue or aberrant vessels. In cases where pre-operative imaging showed evidence of nephrolithiasis, an intraoperative nephroscopy with stone extraction was performed. A flexible cystoscope inserted through one of the trocars can allow, with the TilePro function, simultaneous manipulation and visualization of robotic and endoscopic inputs. Stones were withdrawn without lithotripsy using a Nitinol basket. In one case, the patient with a duplex, malrotated kidney was found to have intrinsic UPJO of the upper pole moiety and crossing vessel obstruction of the lower pole pelvis. The surgical correction was upper pole to lower pole pelvis side to side pyelo-pyelostomy anastomosis and separate dismembered pyeloplasty of the lower pole ureter to the lower pole pelvis (Fig. 1). In two cases where high insertion was found to be the underlying etiology of UPJO, Foley Y-V plasty was performed using standard technique [10]. In all cases, a double-J stent was placed prior to completing the anastomosis. After placing the posterior sutures, an indwelling ureteral stent was placed. The anastomosis was then pressure tested by injecting saline using a cyst aspiration needle through the renal pelvis. Interceed ® [Ethicon, Neuchatel, Switzerland.] anti-adhesion material was then placed around the ureter and renal pelvis to prevent entrapment of the ureter in the fibrotic reaction.

A 19 French Blake drain was placed in the perinephric position in all patients. The ureteric stent was removed after 4–6 weeks. A follow up renogram was obtained 3–6 months after surgery. Success was defined as symptom resolution and/or improvement in radiologic evidence of obstruction. Post-operative complications were classified according to the Clavien–Dindo grading system.

## Results

**Fig. 2:**
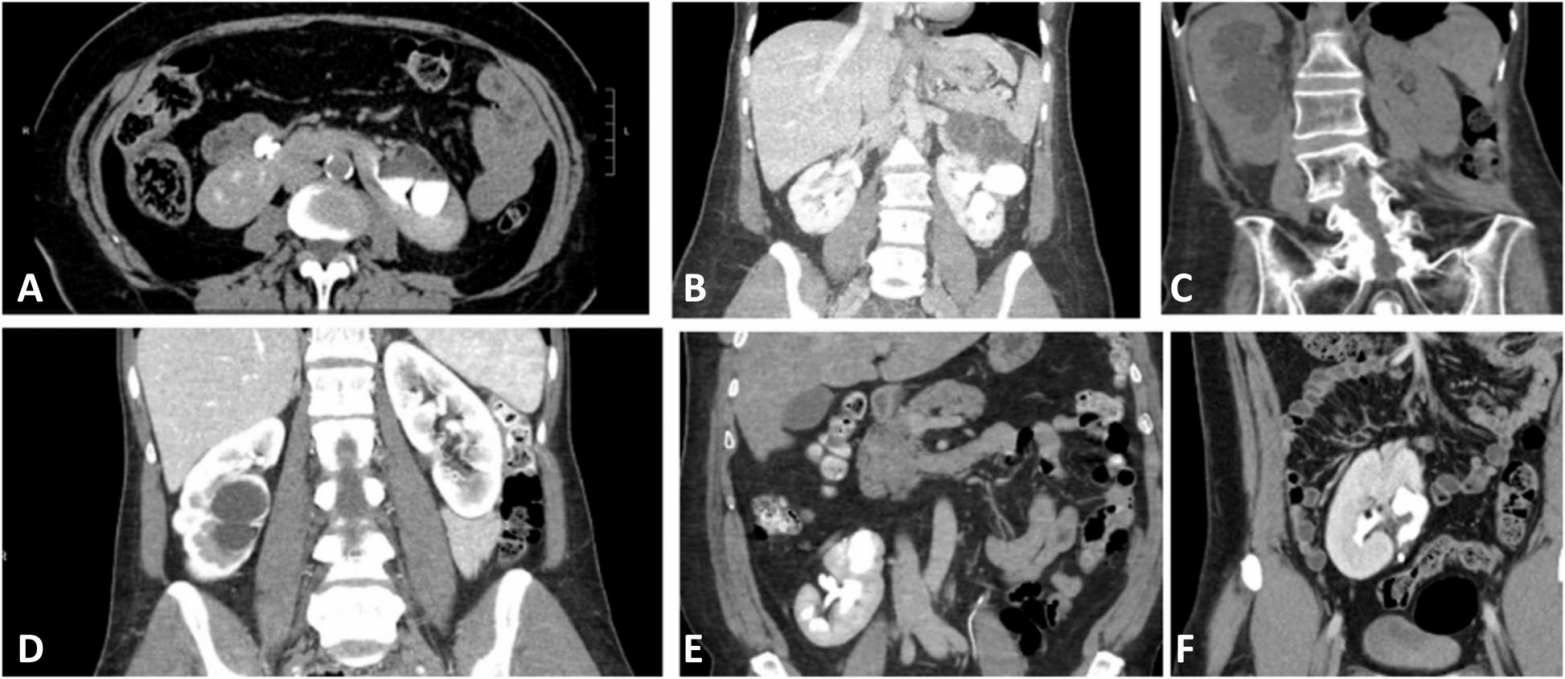
CT demonstrating various congenital renal anomalies: **A** Horseshoe kidney with left UPJO, **B** Duplex malrotated left kidney with upper pole hydronephrosis, **C** Right malrotated kidney, **D** Right sided duplicated system with lower pole hydronephrosis, **E** Ptotic malrotated left kidney, **F **Left pelvic kidney

During the study period, a total of ten patients ( 4 female, 6 male) with a mean age of age of 47 ± 15 years were identified as having simultaneous congenital renal abnormalities and UPJO. The most common abnormality identified was horseshoe kidney in 4 patients followed by duplex kidney in two patients, malrotated kidney in two patients and pelvic kidney in two patients (Fig. 2). Most of the patients (8/10) were symptomatic at presentation with flank pain being the most common symptom. Preoperative work up included CT scan and Lasix renogram. Renogram was omitted in one patient who already had a stent in place. The mean operative time was 160 ± 20 min with a mean blood loss of 50 cc ± 47 cc. Eight patients underwent robotic pyeloplasty via the dismembered technique, while the other two underwent robotic Y-V pyeloplasty. The urethral catheter was removed on the following day. Seven patients (7/10) were discharged on postoperative day one, two patient was discharged on day two POD2 and one with a pelvic kidney who developed urine leak was discharged after four days with the drain. All patients were discharged without an indwelling drain.

 Over a mean follow up time of 13 months, 8/9 symptomatic patients had resolution of symptoms. One patient with a ptotic and malrotated kidney did not experience symptom resolution. The patients’ symptoms persisted despite two subsequent laser endopyelotomy procedures. Of note, this patient did have decreased renal function but did not have imaging evidence of obstruction in preoperative renogram (T ½: 8 min). A retrograde pyelogram was performed which showed a narrowing in the UPJ. Two patients were asymptomatic, although Lasix renogram was consistent with obstruction. One patient with the pelvic kidney (28-year-old in Table 1) was asymptomatic in terms of flank pain before and after surgery. Follow up imaging for the pelvic left kidney revealed a T1/2 of 20 min and severe hydronephrosis.

Post-op renogram were available for nine patients and showed no evidence of obstruction in all patients (Table 1).

**Table 1:**
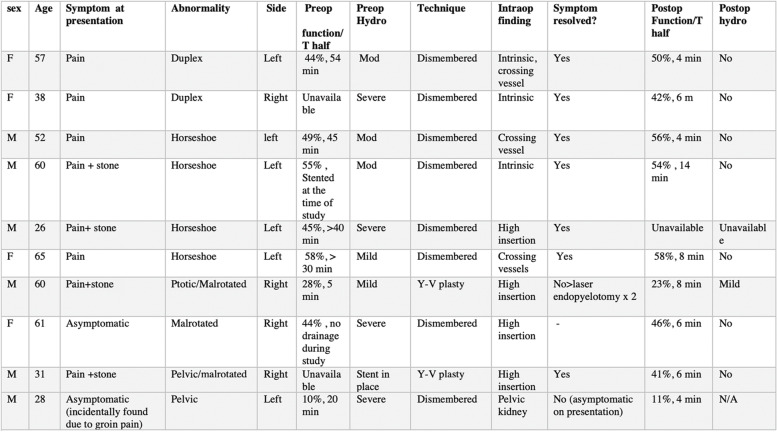
Characteristics of patients with renal anomalies and UPJO who underwent robotic assisted laparoscopic pyeloplasty

There were no intraoperative complications or conversions to open surgery. The patient with a pelvic/malrotated kidney was found on POD 1 to have increased drain output consistent with urine leak which was managed by keeping the drain in place for extra three days with resolution during that time.

## Discussion

Robotic assisted laparoscopic pyeloplasty is associated with a high success rate and low complication rate in adult population. It is considered the standard of care for a high success rate and reduced morbidity compared to open techniques [11]. Little has been published regarding the safety and efficacy of minimally invasive pyeloplasty in adult patients with congenital renal abnormalities [5, 12]. Distorted anatomy, severe hydronephrosis, kidney stones and ptosis of the kidney are thought to affect the healing process in UPJO repair [13]. In the study by Nayyar et al., Patients had a combination of these complicating factors; however, clinical success, radiologic success, operative time, estimated blood loss and hospital stay were comparable RAP in patients with normal anatomy [12, 14]. All symptomatic patients with evidence of obstruction on preoperative renogram became asymptomatic with resolution of obstruction on post op renogram.

In our cohort, only one patient with ptotic malrotated kidney did not experience symptomatic relief despite pyeloplasty and two subsequent endoscopic treatments. Fontenot et al., reported that patients with equivocal preoperative renogram are less likely to experience symptomatic relief following pyeloplasty [15]. Even in patients with normal anatomy, persistent of symptoms without objective evidence of UPJO remains a challenge for urologists. Lee et al. published their experience on 19 cases with recurrent symptoms following pyeloplasty. A fifth of patients ended up with chronic flank pain despite endoscopic treatment without objective evidence for secondary UPJO and were ultimately referred to a pain specialist [16].

A horseshoe kidney is a renal fusion anomaly characterized by the aggregationof the two kidneys over the spine across the midline. Its incidence is 1 in 400–1000 births, with a slight male predominance. It is usually asymptomatic but concomitant UPJ obstruction in 15–33% of patients can alter the clinical course. Multiple techniques have been described in the literature regarding robotic pyeloplasty in patients with horseshoe kidney; some of them include dividing the isthmus [6]. In our series, all of our patients with horseshoe kidney underwent dismembered pyeloplasty with subsequent resolution of symptoms. The isthmus was not divided as this did not appear to influence the obstruction. Two patients with a horseshoe kidney were followed for 4 and 6 years, respectively showing durable effects which is one of the longest follow ups reported for this subset of patients [17].

Urinary tract duplication is the most common anomaly of the upper urinary tract with a reported incidence of 0. 8%. They can be complete or incomplete and are associated with vesicoureteral reflux, ureterocele, and ectopic ureter [18]. UPJO occurs in 2–7% of patients with duplex kidneys and usually involves the upper moiety. Depending on the anatomy, various techniques of robotic UPJO repair in partial duplex kidneys is reported in the pediatric population. Options include lower pole dismembered pyeloplasty, end to side, end to end uretero-pyelostomy, and upper pole partial nephrectomy [8, 9, 19]. In our patient with partial duplication, the upper moiety ureter proximal to the confluence was short and stenotic; the decision was made to excise that segment and perform pyelopyelostomy. Sahai et al., reported a similar case of a 41-year-old adult laparoscopic pyeloplasty and pyelopyelostomy for UPJO at the level of the confluence [20]. Ureteral duplication is a highly variable anatomy and therefore, individualized surgical approach should be considered based on preoperative imaging and intraoperative findings.

The development of a pelvic kidney occurs due to an error during the normal ascension process in the ninth week of fetal development that causes the kidney to remain in the pelvis [21]. Uteropelvic junction obstruction occurs in 22–37% of pelvic kidneys making it a very rare entity [22]. Nuclear imaging in pelvic kidneys is challenging, as posterior imaging is often the only angle captured and can underestimated renal function Due to the pelvis acting as a barrier between the gamma camera and the radioactive tracer. Allen et al., followed a patient with 46% renal function in pelvic kidney. Three years later, posterior imaging showed 20% reduction in renal function. Repeat imaging with posterior and anterior imaging confirmed renal function consistent with baseline [23]. Therefore, it is essential to obtain both anterior and posterior images for better renal function assessment in pelvic kidney patients. In our series, a 28-year-old patient with incidentally discovered pelvic kidney, was reported to have 10% renal function on preoperative and 11% on postoperative renogram. Review of the postoperative renogram revealed that only posterior images were captured, possibly underestimating renal function.

Surgeon experience is of paramount importance in robotic reconstructive surgery in patients with rare challenging anatomy. Learning curve for robotic pyeloplasty in anatomically normal cases has been studied in pediatric urology and is reported to be between 20 and 40 cases [24, 25]. The surgeon in this study had exceeded this number of cases before this series.

To our knowledge, this is the first series on robotic-assisted laparoscopic pyeloplasty in adults with a wide range of renal abnormalities. Anomalies identified were horseshoe kidney in 4 patients, duplex kidney with obstruction of one moiety in two patients, malrotated kidney in two patients, and pelvic kidney in two patients. The overall radiologic resolution of obstruction was 100% and symptom resolution was achieved in 8/9 [89%] of our symptomatic patients. The success rate including radiologic success and symptom resolution as well as operative time, blood loss and complication rate compare favorably to the literature with RAP in otherwise normal kidneys [7]. The results of this study should be considered given its limitations including its retrospective, non-randomized nature and the heterogeneous patients’ cohort. However, given the rarity of the conditions, we believe this study will expand the literature on the efficacy and safety of robotic pyeloplasty.

## Conclusion

In conclusion, our study suggests that robot-assisted laparoscopic pyeloplasty for ureteropelvic junction obstruction in patients with renal anatomic abnormalities is a safe and feasible option in experienced hands.

## Data Availability

The datasets generated during and/or analyzed during the current study are available from the corresponding author on reasonable request.

## Abbreviations

UPJO: Ureteropelvic junction obstruction
RAP: Robotic assisted laparoscopic pyeloplasty
MAG3: Mercaptoacetyltriglycine
